# Stability indicating eco-friendly quantitation of terbutaline and its pro-drug bambuterol using quantitative proton nuclear magnetic spectroscopy

**DOI:** 10.1186/s13065-024-01120-7

**Published:** 2024-01-30

**Authors:** Asmaa El-Sayed, Mohamed A. Sabry, Heba Elmansi, Manal Eid, Shereen Shalan

**Affiliations:** Department of Pharmaceutical Analytical Chemistry, Faculty of Pharmacy, Mansoura University, Mansoura, 35516 Egypt

**Keywords:** Terbutaline, Bambuterol, NMR spectroscopy

## Abstract

Quantitative ^1^H-NMR became an increasingly important issue in pharmaceutical analytical chemistry. This study used NMR spectroscopy to assay the bronchodilator drug terbutaline sulfate and its pro-drug bambuterol hydrochloride in pure form and pharmaceutical preparations. The technique proceeded using deuterium oxide (D_2_O) as an ^1^H-NMR solvent and phloroglucinol anhydrous as an internal standard (IS). Comparatively, to the phloroglucinol signal at 5.9 ppm, the resulting quantitative signals of the studied drugs were corrected. The terbutaline singlet signal at 6.3 ppm was chosen for quantification, while the bambuterol quantitative singlet signal was at 2.9 ppm. The two drugs were rectilinear over the concentration range of 1.0–16.0 mg/mL. LOD values were 0.19 and 0.21 mg/mL while LOQ values were 0.58 and 0.64 mg/mL for terbutaline and bambuterol respectively. The developed method has been validated according to the International Conference of Harmonization (ICH) regarding linearity, accuracy, precision, specificity, and robustness. A greenness profile assessment was applied, and the method proved to be green. The method enables the assay of the two drugs in pure drug and pharmaceutical preparations. The method also enables the assay of the two drugs in the presence of each other; thus, it is considered a stability-indicating method where terbutaline is an acid degradation product of bambuterol.

**Supplementary Information:**

The online version contains supplementary material available at 10.1186/s13065-024-01120-7.

## Introduction

**Fig. 1:**
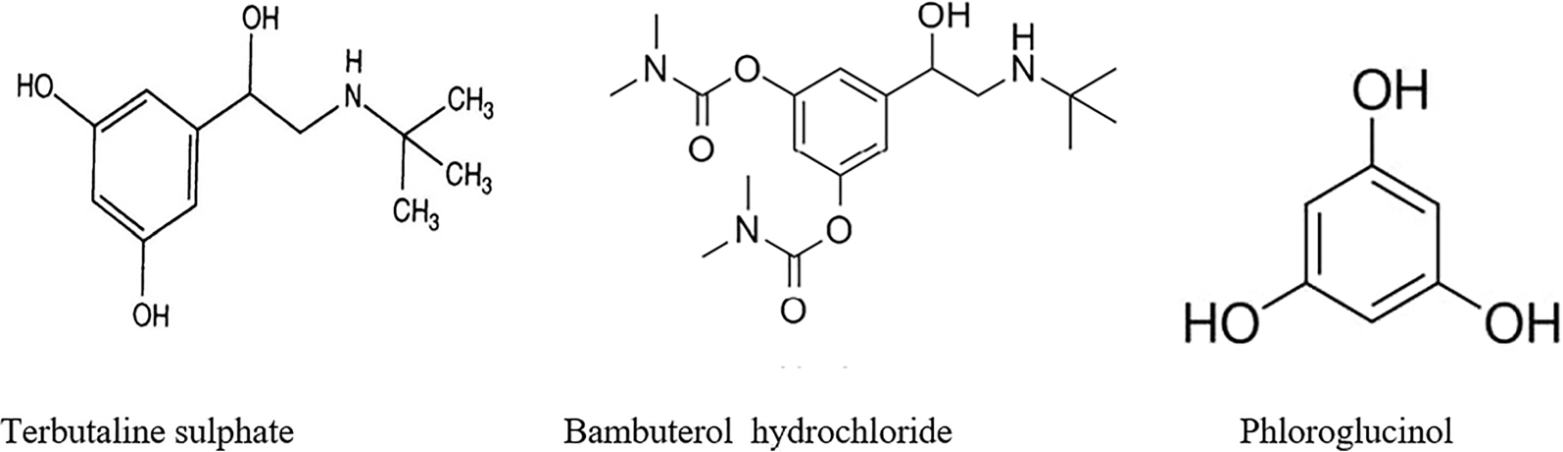
Chemical structures of the studied drugs and the corresponding internal standard

Nowadays, respiratory diseases are among the most serious problems threatening the global community. Bronchial asthma is a chronic respiratory disease that needs ongoing medication. Bronchodilators are among the drugs controlling bronchial asthma [1]. Terbutaline is a bronchodilator that has a stimulant effect on β2-adrenergic receptors of bronchial smooth muscle allowing stability of asthma symptoms [2]. It is chemically 5-[2-(tert-butylamino)-1-hydroxyethyl] benzene-1,3-diol (Fig. 1). Bambuterol is a pro-drug of terbutaline; chemically named 5-[2-(tert-butylamino)-1-hydroxyethyl] benzene-1,3-diol (Fig. 1). Bambuterol provides long-acting bronchodilator with minor adverse effect [3]. Both drugs are official in the British (BP) [4] and United States (USP) [5] Pharmacopeias. Terbutaline is the active metabolite and the acid degradation product of bambuterol. As revealed by the literature, several research papers assayed each of the drugs alone but limited reports assayed them together including; gas chromatography [6], HPLC [7], liquid chromatography with mass spectroscopy [8, 9], the ratio-spectra method [10] and synchronous spectrofluorimetric [11] methods.

Nuclear magnetic resonance spectroscopy (NMR) is widely used in pharmaceutical analysis to identify a drug and the accompanying impurities; to assess the level of impurities, to explain their structure and/or to observe the course of degradation resulting in the related impurities.

Nuclear magnetic resonance spectroscopy (NMR) is a highly significant analytical tool either for identification or quantification purposes. Because NMR observes the nuclear spin of atoms of the molecules, it provides a very specific tool for the determination of the concentration and purity of pharmaceutical compounds [12]. This technique is considered a widespread quantitative analytical technique. In the pharmaceutical field, applications of quantitative NMR (qNMR) cover mostly drug quantification and biological metabolites offering an unbiased view of the sample composition, and the possibility to simultaneously quantifying multiple compounds. Though the sensitivity of NMR assay is lower than that of HPLC or mass spectroscopy [13], it has several advantages, including the capacity to analyze multicomponent mixtures without prior isolation, the capability to provide information on the chemical structure, and the ability to assay different samples without causing damage to them [14, 15]. Quantitative NMR (qNMR) can be used to estimate a molecule's concentration in solution by using information from a nuclear magnetic resonance spectrum, specifically the integral signal of an NMR peak. The integral signal depends on the number of nuclei in the relevant signal, which in turn depends on the substance concentration. Proton NMR (^1^H-NMR) is the most common type of NMR due to the high abundance of hydrogen in nature but other nuclei such as carbon C13, phosphorus P31, fluorine F19 can be used. 1H-qNMR was utilized in the assay of many drugs in pure form and in their dosage form like clindamycin [15] using potassium hydrogen phthalate as the internal standard and deuterium oxide (D2O) as the NMR solvent and memantine [16] using caffeine as an internal standard and D2O as the NMR solvent. Other drugs including: tadalafil [17], favipiravir [18] sofosbuvir, ledipasvir, and daclatasvir [19] were also determined. Additionally, research papers have reported the use of 1H-NMR in the determination of co-formulated drugs or related pharmaceutical compounds like an assay of aspirin, phenacetin and caffeine [20], an assay of levofloxacin, metronidazole and sulfamethoxazole [21] and analysis of fluticasone propionate and azelastine hydrochloride [22] in their combined pharmaceutical preparations. Subsequently, International Pharmacopoeias [4, 5] reported qualitative NMR spectroscopy for identification purposes and quantitative NMR spectroscopy to evaluate the composition of polymers and impurities in drugs.

Up to now, quantitative NMR (qNMR) measurements are at least as reliable and precise as those attained by the more frequently used chromatography-based procedures, while allowing various advantages including simple method development, easy sample preparation and relatively short analysis times.

No method was reported in the literature for the assay of terbutaline sulphate in the presence of its pro-drug bambuterol hydrochloride using ^1^H-qNMR. The proposed method assayed terbutaline together with its pro-drug bambuterol by ^1^H-qNMR. The method is considered green, rapid, specific, and sensitive technique for the determination of terbutaline and bambuterol singly in their pharmaceutical formulations and in the presence of each other in stability-indicating tests.

## Experimental

### Materials and methods

- Terbutaline was brought from Sedico, Egypt, with a purity of 99.97% as labeled by the company.
- Bambuterol was brought from AstraZeneca Pharmaceutical Egypt. The purity was found to be 99.98% as informed by the manufacturer.
- Phloroglucinol anhydrous (PHL, 99.8% purity) as an internal standard was brought from Chemipharm for Pharmaceutical Industries, 6th October City, Giza, Egypt.
- Deuterium oxide (D_2_O) as q- ^1^HNMR solvent was brought from Memphis for Pharmaceutical and Chemical Industries, Cairo, Egypt.
- Aironyl® tablet contains 2.5 mg terbutaline, produced by SEDICO, 6 October City, Egypt.
- probric® tablet contains 10 mg bambuterol produced by Borg Pharmaceutical IND., Alexandria, Egypt.

### Instrumentation

- All ^1^HNMR experiments were carried out using Bruker 400 MHz Avance III spectrometer under the following acquisition conditions: spectral width (15 ppm), frequency offset (6.175 ppm), acquisition time (4.08 s), pulse angle (10°), pulse width (1.5 µs), sample temperature (20°C), dummy scans (2), relaxation delay (10 s), number of scans (32), sample spin (on) and data points (65536)
- For the comparison method [10], the Cary Eclipse fluorescence spectrophotometer from Agilent Technologies and Cary Eclipse software were used for measuring synchronous fluorescence spectra at ∆λ = 20 nm, smoothing factor = 15 and slit width = 5 nm during all the experiments. The instrument was adjusted at high sensitivity mode (800 v).
- EUMAX-3L-3-4Gl-Heated Digital Ultrasonic bath, Model: Soner 206 H, MTI Corporation, USA was utilized when sonication is needed.

### Preparation of standard solutions

To make a standard solution from each drug: 20.0 mg of each of the studied drugs and 10.0 mg of anhydrous phloroglucinol were dissolved in 1.0 mL D_2_O separately in stoppered glass vials to obtain a final concentration of 20.0 mg/mL for the two drugs and 10.0 mg/mL solution of phloroglucinol. Then all the solutions were sonicated. The two drugs' serum concentrations were prepared by diluting the standard solutions to obtain a concentration range of 1.0-16.0 mg/mL for each drug. Phloroglucinol internal standard was added with a final 10.0 mg/mL concentration. The volume was completed to 1.0 mL then aliquots of the working solutions were then transferred to NMR tubes and measured in triplicate for each concentration under the estimated conditions. Absolute integral areas were plotted against corresponding concentrations and regression equations were plotted.

### Preparation of tablet solution

Ten tablets of each drug preparation were pulverized then the amount equivalent to one tablet of each drug was weighted and extracted with 2.0 mL D_2_O by sonication. Solutions were filtered and the procedure mentioned under –preparation of standard solution- was followed.

### Calculation of the relaxation time:

The main factor affecting qNMR quantitation is the relaxation delay time. It depends on the aggregated longitudinal relaxation time (T_1_) of the responses over all acquisitions. T_1_ may be computed using the formula that follows:

$${\text{M}}_{{\text{z}}} = {\text{ M}}_{0} \,\left( {{1}{-}{\text{e}}_{1}^{{^{{ - \left( {\tau \, /{\text{T}}} \right)}} }} } \right)$$

Where: The z-axis magnetizations are Mz and M_0_. The repetition period during which the spin-lattice relaxes and reaches thermal equilibrium is denoted as τ. To ensure the validity of the experimental results gathered, T should be at least five times T_1_ [23].

### Theory

The primary foundational principle of qNMR is that the integrated signal area (I) is directly proportional to the number of nuclei (n), resulting in the conforming line of resonance as follows:

$$I = Ks \times n$$

where: n denotes the relative number of spins (protons) that produce the resonance, and K_s_ is a constant associated with each spectrometer [24]. Calculating the relative area ratio I_1_/I_2_ is the best way to certify quantitative results [17] because K_s_ is constant and can be omitted leading to the following equation:

$$I1/I2 = n1/n2$$

The following formulae can be used to calculate the purity of a drug using the NMR in the presence of a known internal standard, based on this equation:

$$\mathrm{Wx }=Ix. Nstd. Mx. mstdIstd. Nx. Mstd$$

$$\mathrm{Px }={\text{Ix}}.\mathrm{ Nstd}.\mathrm{ Mx}.\mathrm{ mstd}.\mathrm{ Pstd}/{\text{Istd}}.\mathrm{ Nx}.\mathrm{ Mstd}.\mathrm{ m}$$

where: W_x_ and P_x_ are the weight and pureness of the studied drug, correspondingly. M_x_ and M_Std_ are the molecular weights of the studied drug and the internal standard, correspondingly. I_x_ and I_Std_ are the integrated peak area of the studied drug and the internal standard, correspondingly. N_x_ and N_Std_ relate to the number of protons in the integrated peak area of the studied drug and the internal standard, whereas m and m_Std_ are the weight of the studied drug and the internal standard correspondingly and P_std_ is the pureness of the internal standard.

## Results and discussion

Development of the quantitative ^1^H-NMR technique necessitate presence of distinguished signals of the studied drugs and the internal standard. qNMR analysis is often more accurate and precise than standard HPLC methods, no isolation of the impurity is necessary, and no expensive chemical reference substances are necessary. Singlet signals are preferred in quantification as they are more accurate. Though terbutaline and bambuterol are chemically related compounds, they show separated singlet signals at 6.3 ppm and 2.9 ppm respectively. Phloroglucinol internal standard was used in our technique as its singlet quantitative protons were recorded at 5.9 and 8.9 ppm with no interference with signals of the analyzed drugs (Additional file 1: Figure S1). Thus, our quantitative ^1^H-NMR technique can properly assay terbutaline and bambuterol with phloroglucinol internal standard in pure form, pharmaceutical preparations, and prepared synthetic mixtures. Several critical factors were optimized prior to the assay to increase the selectivity and sensitivity of the method.

### Selection of deuterated solvent

Varied deuterated solvents were tested for proper quantitative assay. DMSO-d6, chloroform-d1, acetone-d6 and deuterium oxide were studied. DMSO-d6 showed poor solubility for the studied drugs. Chloroform-d1 and acetone-d6 were excluded owing to their toxicity and volatility which caused the changeable final volume of the standard and working solutions making quantification impossible. Deuterium oxide was the solvent of choice as it dissolves both drugs, its signal does not interfere with the quantitative signals of the examined drugs and internal standard, it is non-volatile and finally it was a green solvent.

### Selection of internal standard

Several compounds were tested to be used as internal standards. Phloroglucinol was selected as the internal standard for different reasons. Phloroglucinol chemical shift was at 5.9 ppm, so it does not interfere with the chemical shifts of the analyzed drugs. Phloroglucinol also dissolves in deuterium oxide.

### Optimization of NMR parameters

NMR instrumentation is controlled by different parameters which affected the efficacy of the quantification. Such parameters were the number of scans, pulse angle and delay relaxation time. The three factors were adjusted in a systemic matter to produce satisfactory results.

### Number of scans

Different scan numbers were investigated (16, 32, 64, 128) at a pulse angle of 90° and delay time of 1.0 sec. to find the best signal-to-noise ratio. Each experiment was repeated three times and recorded as in (Additional file 1: Figure S2). Scan numbers of 128 and 32 showed the best resolution but scan number of 128 was found to be non-reproducible so scan number of 32 was chosen.

### Pulse angle

Pulse angles of 10, 30, 60 and 90 were investigated at the number of scans of 32 and a relaxation delay time of 1.0 sec. Increasing pulse angle dramatically decreased the sensitivity of the method, so pulse angle of 10° was selected. These data are presented in Additional file 1: Figure S2.

### Delay relaxation time

Relaxation delay time is a critical item affecting the sensitivity of the method. Thus, the relaxation delay time of 1, 5, 10 and 20 was investigated at scan number of 32 and pulse angle of 10°. The ideal delay time for longitudinal relaxation between two consecutive pulses was found to be 10 s, which also provided the best signal resolution and quantitative testing (Additional file 1: Figure S2).

### Validation of the method

The proposed ^1^H-NMR method was validated in accordance with ICH guidelines for the following characters:

### Linearity and range

It was declared before that the intensity of the ^1^H-NMR signal is directly related to the number of nuclei and, therefore, to the drug concentration. Different concentrations of terbutaline and bambuterol ranging from 1.0 to 16.0 mg/mL for the two drugs in deuterium oxide were prepared then the previously mentioned experimental conditions were followed. A linear relationship was observed between drug concentrations and their corresponding absolute integral areas. Data was analyzed statistically [25] and regression equation was plotted. Table 1 demonstrates the linearity of the established approach due to its large correlation coefficient (r >0.999), modest intercepts, standard deviation, and relative error percentage. The calibration curves are illustrated in Additional file 1: Figure S3.

**Table 1:** Analytical performance data for the^1^H-qNMR method

Parameter	Terbutaline	Bambuterol
Linearity rang (mg/mL)	1.0–16.0	1.0–16.0
Limit of detection, LOD (mg/mL)	0.242	0.212
Limit of quantitation, LOQ (mg/mL)	0.732	0.645
Intercept (a)	− 171514.6	− 769676.7
Slope (b)	1575923.5	6976199.7
Correlation coefficient (r)	0.9999	0.9999
S.D. of residuals (S_y/x_)	166853.04	664668.6
S.D. of intercept (S_a_)	115407.44	54488.35
S.D. of slope (S_b_)	12577.02	449982.7
Percentage relative standard deviation, % RSD	1.79	1.68
Percentage relative error, % Error	0.803	0.756

### Limits of detection and quantification

Limits of detection (LOD) and quantification (LOQ) were calculated according to ICH guidelines [26] via the equation of :

$$LOD=3.3\times S_a/b$$

$$LOQ=10\times S_a/b.$$

where S_a_ is the standard deviation of the intercept and b is the slope of the previously constructed calibration curves Table 1. LOD were 0.242 and 0.212 mg/mL and LOQ were 0.732 and 0.645 mg/mL for terbutaline and bambuterol respectively.

### Accuracy and precision

Accuracy is the degree to which the measured value from the developed ^1^H-NMR method and the established comparison method coincide with one other. A spectrofluorimetric method was considered for the comparison of the results together with our proposed method [10]. In the comparison method [10]; the two drugs were assayed at ∆λ =20 by synchronous fluorimetry using water as solvent: terbutaline was measured at 290 nm whereas bambuterol was measured as 260 nm. Three different concentrations of the two drugs were analyzed triplicately, and the percent recoveries were abridged in Table 2. No significant difference was found using student t-test and variance ratio F-test [25], as shown in Table 2.

**Table 2:** Comparative analytical data for the determination of Terbutaline sulphate and Bambuterol hydrochloride in their pure form by proposed ^1^H-qNMR method and a comparison spectrofluorimetric method

Drug	Proposed method			Comparison method ]10[	
Trebutaline mg/ml	Amount taken	Amount found	% Found	Amount taken (µg/ml)	% Found
	1.0	1.0	102.9	0.4	101.5
	2.0	2.0	100.28	0.8	100.63
	4.0	3.92	98.04	1.6	99.06
	12.0	12.0	100.87	3.2	98.84
	16.0	15.93	99.6	4.0	100.85
Mean	100.34			100.73	
± S.D	1.8				
Student´s T-test	0.17 (2.3)*				
Variance F-test	2.32 (5.05)*				
Bambuterol mg/ml	1.0	1.01	101.76	0.2	102.0
	2.0	2.05	102.67	0.4	98.75
	4.0	4.01	100.27	1.6	99.75
	8.0	7.85	98.22	4.0	100.33
	16.0	16.06	100.38	6.0	99.83
Mean	100.66			99.97	
± S.D	1.69				
Student´s T-test	0.57 (2.3)⁎				
Variance F-test	2.01 (5.05)⁎				

Each result is an average of three separate determinations

⁎ The values between parentheses are the tabulated t and F values at P = 0.05 [25]

The repeatability and intermediate precision analyses were confirmed through Intra-day and inter-day assays of different concentrations within the linear range. Repeatability represents precision under the same operating conditions over a short time interval while intermediate precision as within laboratory variations e.g., different days and different analysts. As revealed in Table 3, small values of relative standard deviations confirmed good precision for the proposed NMR method.

**Table 3:** Reproducibility and precision data for the analysis of terbutaline sulphate and bambuterol hydrochloride using the proposed ^1^H-qNMR method

Drug	Conc.	Intra-day			Conc.	Inter-day		
		% error	% RSD	Mean ± S.D		% error	% RSD	Mean ± S.D
Terbutaline	2.0	0.15	0.26	101.8 ± 0.27	2.0	0.64	1.12	98.73 ± 1.10
	4.0	1.09	1.89	100.07 ± 1.89	4.0	0.34	0.59	98.33 ± 0.58
	6.0	0.10	0.18	98.1 ± 0.17	12.0	0.97	1.68	99.93 ± 1.68
Bambuterol	2.0	0.15	0.26	101.8 ± 0.27	2.0	1.59	2.76	99.95 ± 2.76
	4.0	1.02	1.76	99.25 ± 1.75	4.0	1.1	1.91	100.65 ± 1.92
	8.0	1.12	1.95	101.03 ± 1.97	16.0	0.97	1.69	100.08 ± 1.69

### Specificity and selectivity

The specificity of our ^1^H-NMR method was confirmed by individually investigating spectra of deuterium oxide (solvent), phloroglucinol internal standard, pure drugs, and sample solutions. Separate signals of the studied drugs were found with no interference with each other or with the internal standard as shown in Additional file 1: Figure S1. Terbutaline singlet signal was recorded at 6.3 ppm, bambuterol singlet signal at 2.9 ppm and phlorogucinol internal standard singlet signal at 5.9 ppm, which confirmed method specificity. The studied drugs were assayed in tablet dosage form with no interference with tablet additives which confirmed method selectivity (Table 4).

**Table 4:** Determination of the investigated drugs in their pharmaceutical tablet dosage forms using the proposed and comparison methods

Compound	Proposed method			Comparison method [10]	
Aironyl^®^2.5 mg terbutaline/ml	Amount taken	Amount found	% Found	Amount taken (µg/ml)	% Found
	1.0	1.02	102.0	1.25	101.76
	1.5	1.47	98.0	2.5	98.25
	2.0	1.96	101.5	4.0	100.59
Mean	100.5			100.2	
± S.D	1.8				
Student´s T-test	0.18 (2.7)⁎				
Variance F-test	1.48 (19.0)⁎				
Probric^®^ tablet 10 mg bambuterol	1.0	0.98	98.0	0.2	102.0
	2.0	1.992	99.6	0.4	98.75
	4.0	3.91	98.0	1.6	99.75
Mean	98.5			100.39	
± S.D	1.7				
Student´s T-test	1.2 (2.7)⁎				
Variance F-test	6.3 (19.0)⁎				

Each result is an average of three separate determinations

^⁎^The values between parentheses are the tabulated t and F values at P = 0.05[25]

### Robustness

The robustness of the ^1^H-NMR method was confirmed by studying minor changes in method parameters. Minor changes in the following acquisition parameters: number of scans, relaxation delay time, pulse angle, data points, spectral width and acquisition time were scanned. No significant difference in the results was found which confirmed method robustness.

### Assay of the studied drugs in their tablet formulations and in prepared synthetic mixtures

The developed qNMR technique was used in the assay of the studied drugs in their tablet dosage forms and their prepared synthetic mixtures with perfect specificity and selectivity. Good percent recoveries and minor %RSD proved the specificity of the method in the assay of the two drugs in the presence of tablet excipients like: lactose monohydrate, maize starch, povidone, microcrystalline cellulose and magnesium stearate Table 4. The data of the developed NMR method was compared with the comparison synchronous spectrofluorimetric method [10], and no significant difference was found between the two methods using the student t-test and variance ratio F-test as illustrated in Table 4.

### Assessment of green profile

**Fig. 2:**
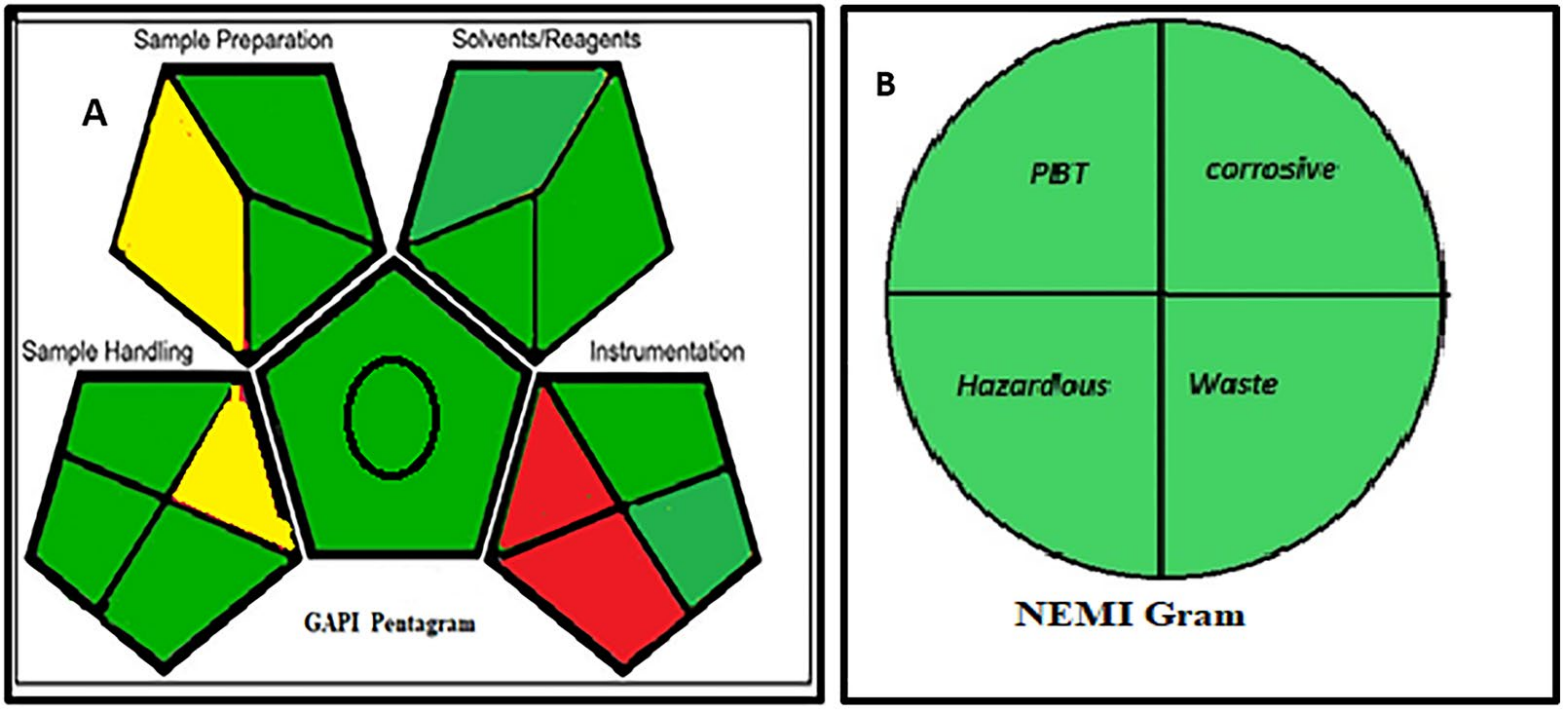
GAPI and the NEMI green profile assessments of the proposed method

Among the community of analysts, there has been a strong concern for the environmental friendliness of any novel analytical strategy. Three tools namely NEMI (National Environmental Method Index), Eco-scale, and GAPI (Green Analytical Procedure Index), were used to assess how environmentally friendly the analytical techniques were. NEMI, one of the earliest methods for measuring greenness, operates under the following four guiding principles: PBT (solvent persistency, bioaccumulation, and toxicity), hazardous effect (use of compounds on the D, F, P, or U lists for being caustic, flammable, reactive, toxic, or extremely), corrosive impacts (pH of 2 to 12), and the amount of waste produced (less than 50 g per sample) [26]. Our quantitative ^1^H NMR method met each of the previously listed requirements for being green which was clarified in Figure 2. The Eco-Scale technique involves subtracting from a base of 100 and determining penalty points for the utilized solvents and other process parameters [27]. The proposed NMR technique had an Eco score of 98, indicating their greenness as shown in Table 5. GAPI is a visual presentation approach consisting of five pentagrams. The impacts of the technique's key steps (sample collection, preservation, transportation, and storage, sample collection, preparation, reagents, chemicals employed, and instruments) are marked red, yellow, and green colors to indicate whether the hazardous consequences are high, medium, or low [28]. Most method pentagrams were green except for waste treatment and the instrumentation which possesses energy more than 1.5 kWh per sample, were red (Figure 2).

**Table 5:** Greenness evaluation for the proposed method using Analytical Eco-scale [27]

Reagents/technique	Proposed method
Deuterated H_2_O	**0**
INRTRUMENT(NMR)	**2**
Waste › 1 ml	**0**
Occupational hazard	**0**
Total penalty points	**2**
Analytical Eco-Scale total score	**98**

## Conclusion

It was anticipated that ^1^H-NMR would become a quick and straightforward technique for the assay of pharmaceutical active constituents because of its many unique advantages, such as its lack of destructive effects, the fact that the analytes under study do not need to be separated before, and the fact that it does not require the presence of genuine reference standards for the analytes. Terbutaline singlet signal at 6.3 ppm was chosen for quantification while the bambuterol quantitative singlet signal was at 2.9 ppm. The two drugs showed linear response over the concentration range of 1.0-16.0 mg/mL. LOD values were 0.19 and 0.21 mg/mL and LOQ values were 0.58, 0.64 mg/mL for terbutaline and bambuterol, respectively. The proposed ^1^H-NMR method allows eco-friendly, simple, accurate, sensitive, and rapid assay of terbutaline sulphate and its pro-drug bambuterol hydrochloride. Phloroglucinol was used as an internal standard in the assay, which was run on deuterium oxide. Following ICH criteria, the method was verified and determined to be linear, accurate, precise, sensitive, and to have acceptable ranges for LOD and LOQ. The method allowed the determination of the two drugs in their pharmaceutical preparations and in the presence of each other in prepared synthetic mixtures, which allowed the use of the method of stability indication.

### Supplementary Information

**Additional file 1: Figure S1.** **a** 1H-NMR spectrum of Phloroglucinol anhydrous in deuterated water (D_2_O). **b** 1H-NMR spectrum of Terbutaline Sulphate at 6.3 ppm with Phloroglucinol anhydrous 5.9 ppm in D_2_O. **c** 1H-NMR spectrum of Bambuterol hydrochloride at 2.9 ppm with Phloroglucinol anhydrous at 5.9 ppm in D_2_O. **Figure S2.** Effect of number of scans on the absolute integral area of selected signals of the two drugs in ^1^H-NMR. **A** Effect of pulse angle on the absolute integral area of selected signals of the two drugs in ^1^H-NMR. **B** Effect of relaxation delay time on the absolute integral area of selected signals of the two drugs in ^1^H-NMR. **Figure S3.** The calibration curves for the determination of a- terbutaline and b- Bambuterol using the proposed NMR method. **Figure S4.** ^1^H-NMR spectrum of 4.0 mg/ml terbutaline sulphate and 10.0 mg/ml bambuterol hydrochloride in their prepared laboratory mixtures using 10.0 mg/ml phloroglucinol IS and D_2_O as solvent.

## Data Availability

Data will be made available upon direct reasonable request from the corresponding author.

## Abbreviations

1H-NMR: Proton Nuclear Magnetic Resonance
D_2_O: Deuterated water
IS: Internal standard
LOD: Limit of detection
LOQ: Limit of quantification
ICH: International conference of harmonization
BP: British pharmacopiea
qNMR: Quantitative nuclear magnetic resonance
T_1_: Relaxation time
M_z_: Z-axis magnetization
M_0_: Z-axis magnetization
n: Number of nuclei
K_s_: Constant related to spectrometer
I: Integrated signal area
W_x_: Pureness of studied drug
P_x_: Pureness of internal satandard
M_x_: Molecular weight of studied drug
M_std_: Molecular weight of internal standard
I_x_: Integrated peak area of studied drug
I_std_: Integrated peak area of internal standard
N_x_: Amount of protons in the integrated peak area of studied drug
N_std_: Amount of protons in the integrated peak area of internal standard
P_std_: Pureness of internal standard
m: Weight of studied drug
m_std_: Weight of internal standard
S_a_: Standard deviation
b: Slope
τ: The repetition period during which the spin–lattice relaxes and reaches thermal equilibrium
NEMI: National Environmental Methods Index
GAPI: Green Analytical Procedures Index
