# Supplementary material for: Stability indicating eco-friendly quantitation of terbutaline and its pro-drug bambuterol using quantitative proton nuclear magnetic spectroscopy
Source: BMC Chem. 2024 Jan 30;18(1):25. doi: 10.1186/s13065-024-01120-7 (PMC10829239; doi:10.1186/s13065-024-01120-7)
Supplement: Supplementary file 1 — Additional file 1: Figure S1. a 1H-NMR spectrum of Phloroglucinol anhydrous in deuterated water (D2O). b 1H-NMR spectrum of Terbutaline Sulphate at 6.3 ppm with Phloroglucinol anhydrous 5.9 ppm in D2O. c 1H-NMR spectrum of Bambuterol hydrochloride at 2.9 ppm with Phloroglucinol anhydrous at 5.9 ppm in D2O. Figure S2. Effect of number of scans on the absolute integral area of selected signals of the two drugs in 1H-NMR. A Effect of pulse angle on the absolute integral area of selected signals of the two drugs in 1H-NMR. B Effect of relaxation delay time on the absolute integral area of selected signals of the two drugs in 1H-NMR. Figure S3. The calibration curves for the determination of a- terbutaline and b- Bambuterol using the proposed NMR method. Figure S4. 1H-NMR spectrum of 4.0 mg/ml terbutaline sulphate and 10.0 mg/ml bambuterol hydrochloride in their prepared laboratory mixtures using 10.0 mg/ml phloroglucinol IS and D2O as solvent. [file 13065_2024_1120_MOESM1_ESM.docx]

**Figure S1:**

(a) 1H-NMR spectrum of Phloroglucinol anhydrous in deuterated water (D_2_O).

(b) 1H-NMR spectrum of Terbutaline Sulphate at 6.3 ppm with Phloroglucinol anhydrous 5.9 ppm in D_2_O.

(c) 1H-NMR spectrum of Bambuterol hydrochloride at 2.9 ppm with Phloroglucinol anhydrous at 5.9 ppm in D_2_O .

**Figure S2**:

1. Effect of number of scans on the absolute integral area of selected signals of the two drugs in ^1^H-NMR.
2. Effect of pulse angle on the absolute integral area of selected signals of the two drugs in ^1^H-NMR.
3. Effect of relaxation delay time on the absolute integral area of selected signals of the two drugs in ^1^H-NMR

**Figure S3:** The calibration curves for the determination of a- terbutaline and b- Bambuterol using the proposed NMR method.

**Figure S4:** ^1^H-NMR spectrum of 4.0 mg/ml terbutaline sulphate and 10.0 mg/ml bambuterol hydrochloride in their prepared laboratory mixtures using 10.0 mg/ml phloroglucinol IS and D_2_O as solvent.

**
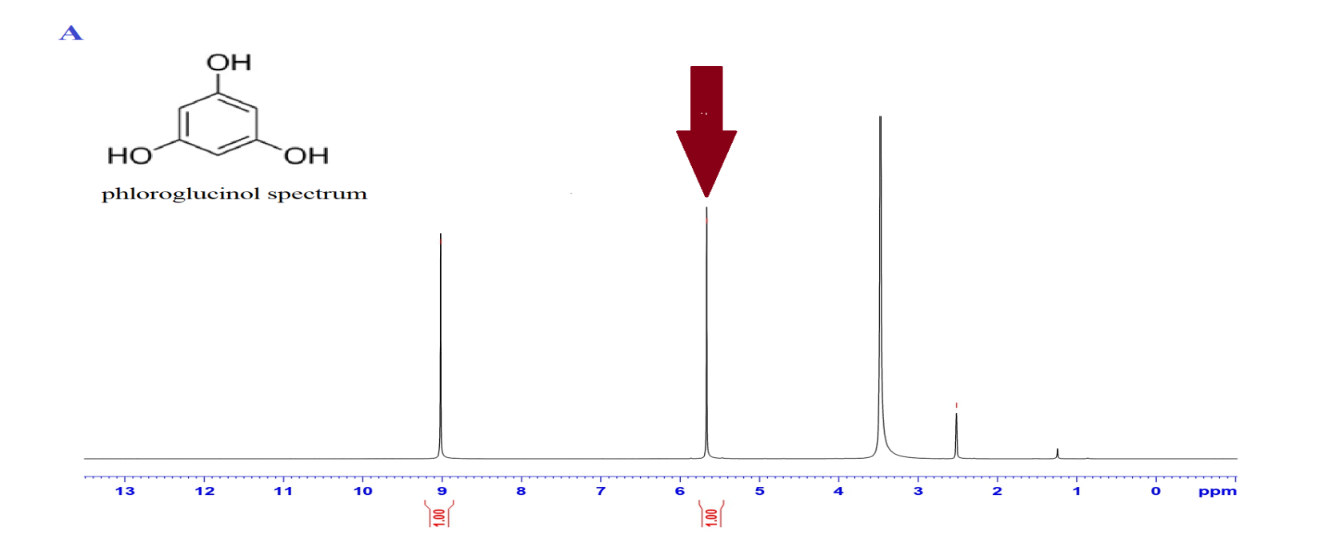

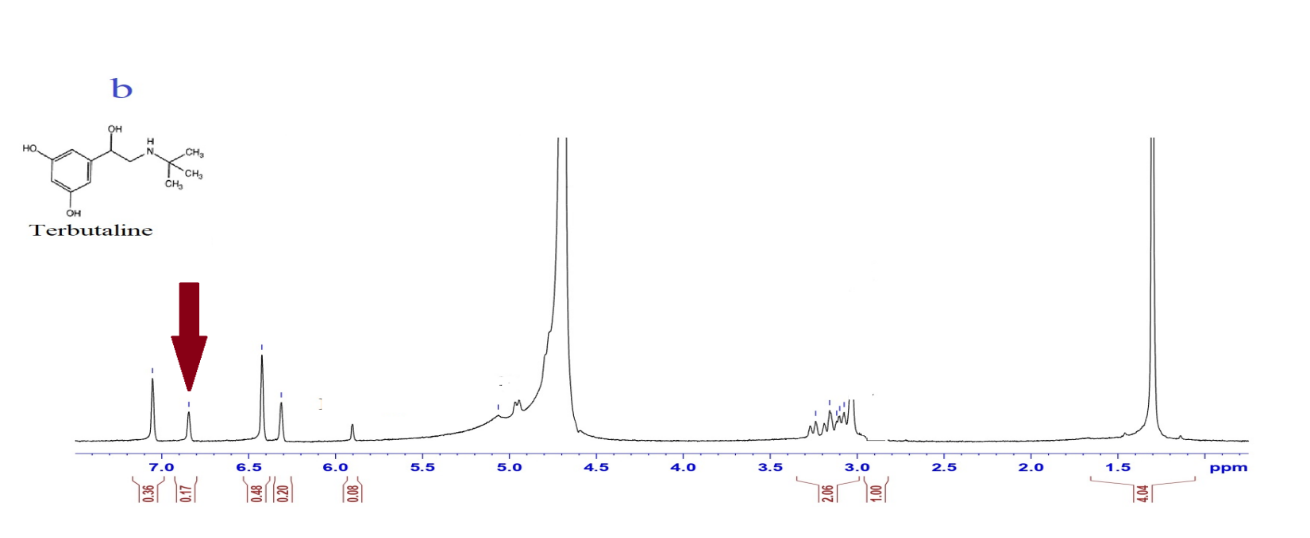
**

**a**

**
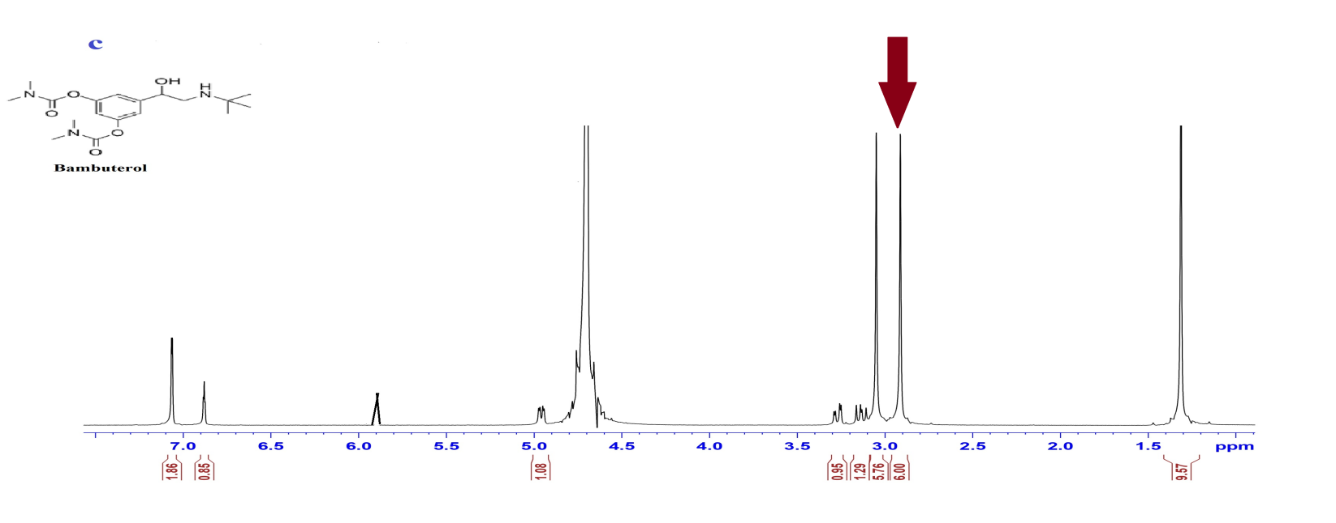
Figure S1**


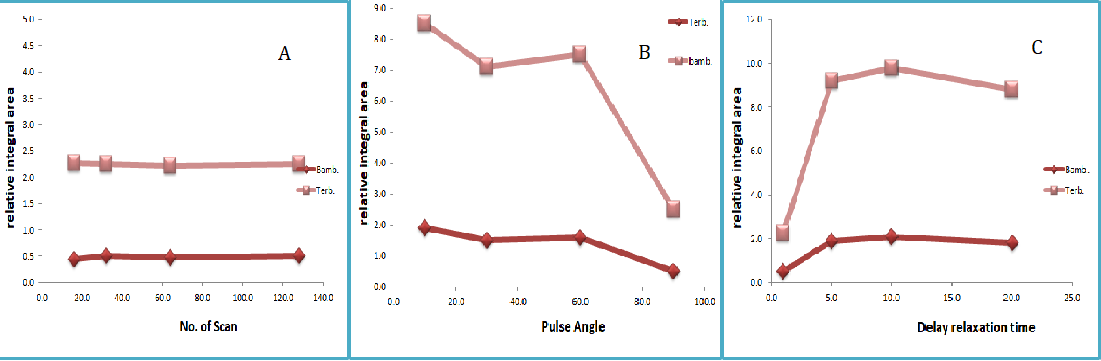


**Figure S2**

**a**

LOD

LOQ

**b**

**Figure S3**

**
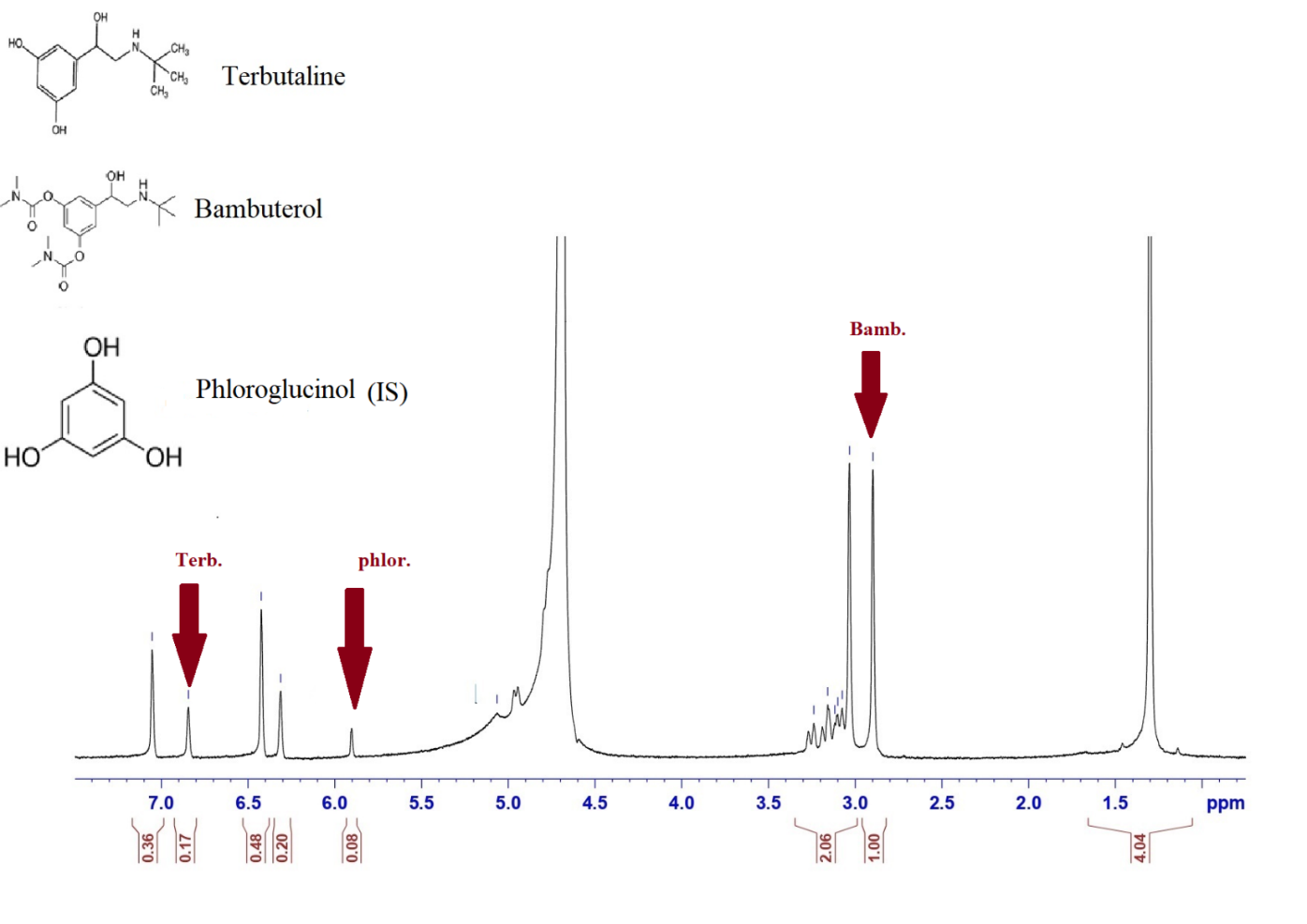
**

**Figure S4**
